# Away with Quacks and Prescribing Chemists

**Published:** 1894-07-14

**Authors:** J. W. Haynes

**Affiliations:** Cuckfield


### Editor's Letter-Box.

[Our correspondents are reminded that proxility is a great bar to publication, and that brevity of style and conciseness of statement greatly facilitate early insertion.]

### AWAY WITH QUACKS AND PRESCRIBING CHEMISTS.

Sir,—Simply as one of the public I beg to place a tribute of thought before abler heads than mine among your readers. Make the sale of medicines and drugs of any kind—for personal purposes—impossible, save to the order of qualified medical men. Only qualified chemists to execute the orders, and then cancel the prescription when it is made up. This would need the appointment of highly able and well paid medical men to examine and prescribe in poor and populous places; they would also have to report on the health of the people to proper authorities. Naturally, this prohibits the sale of all quack medicines. I would also exclude all quack surgery. Chemists having under these terms the exclusive sale of drugs would, perhaps, have no cause of complaint, although it might be well to consider if medical dispensing should not be confined to places where there was no chemist.—I am, sir, yours respectfully,

Cuckfield, July 10th, 1894. J. W. Haynes.

